# Primary cardiac synovial sarcoma that was continuous with the mitral valve caused severe thrombocytopenia: a case report

**DOI:** 10.1186/s13019-019-0852-8

**Published:** 2019-02-04

**Authors:** Guodong Zhang, Qing Gao, Shenglong Chen, Yu Chen

**Affiliations:** Cardiac Surgery Department, Peking University People’s Hospital, Beijing, 100044 China

**Keywords:** Cardiac synovial sarcoma, Thrombocytopenia, Tumour resection

## Abstract

**Background:**

Primary cardiac sarcomas are exceedingly rare, and they commonly result in nonspecific constitutional symptoms such as shortness of breath, weight loss, and anaemia-related fatigue and malaise. However, thrombocytopenia has very rarely been reported in association with cardiac tumours, either benign or malignant. We report one case of primary cardiac synovial sarcoma continuous with the mitral valve, which was accompanied by severe thrombocytopenia, and the platelet counts returned rapidly to a normal range early after tumour excision and without any special therapies.

**Case presentation:**

A 52-year-old male diagnosed with atrial myxoma with severe thrombocytopenia was admitted to our hospital. Blood analysis showed severe thrombocytopenia, whereas erythrocyte and leucocyte counts were within the normal range. A 50 × 35 mm mobile mass continuous with the mitral valve was found to be present in the left atrium upon echocardiography. Bone marrow aspiration and related examinations excluded thrombocytopenia caused by haematologic malignancies. The patient received a platelet transfusion, but platelet counts decreased quickly. Glucocorticoid therapy and immunoglobulin transfusion were also used, but were ineffective. Although the operation risk was high, tumour resection was performed via a median sternotomy with a cardiopulmonary bypass system. The postoperative pathological diagnosis was biphasic cardiac synovial sarcoma. Surprisingly, the platelet counts returned rapidly to a normal range early after tumour excision without any special therapies. The disappearance of the tumour from the annular region was confirmed on transthoracic echocardiography 6 days after surgery, and an FDG-PET scan performed 8 days after surgery showed no abnormal accumulation. Unfortunately, the patient died suddenly 6 months later without unknown cause.

**Conclusions:**

We report that a rare primary cardiac synovial sarcoma case continuous with the mitral valve caused severe thrombocytopenia; this provides further support for the awareness and diagnosis of primary cardiac synovial sarcoma. We also highlight that thrombocytopenia might be one rare symptom of a solid cardiac tumour but need more cases for support.

## Background

Primary malignant tumours affecting the heart are altogether rare, accounting for 5.1%–28.7% of primary cardiac tumours [1]. Primary cardiac sarcomas are extremely rare, consisting of myxosarcoma, intimal sarcoma, synovial sarcoma, liposarcoma, angiosarcoma, fibrosarcoma, etc. [2]. Primary cardiac sarcomas result in nonspecific constitutional symptoms such as shortness of breath, weight loss, and anaemia-related fatigue and malaise [3]. However, severe thrombocytopenia has very rarely been reported in association with cardiac tumours, either benign or malignant [4]. We report one patient diagnosed with atrial myxoma with severe thrombocytopenia on admission, while the postoperative diagnosis was primary cardiac synovial sarcoma (PCSS) that severely adhered to the posterior mitral annulus. Surprisingly, the platelet counts returned rapidly to normal range early after tumour excision without other special therapies. There were few primary cardiac synovial sarcoma cases described in the literature [5], and none of them reported that PCSS can cause severe thrombocytopenia.

## Case presentation

A 52-year-old male presented with paralysis of the left upper extremity; in another hospital 1 year before the current admission, the patient had received a computed tomography (CT) scan, which indicated cerebral infarction. A mass regarded as a myxoma that compressed left atrium was detected by transthoracic echocardiography (TTE), and this was considered to be the cause of cerebral infarction. Blood analysis showed severe thrombocytopenia, whereas erythrocyte and leucocyte counts were at a normal range. Gradually, he developed bilateral lower extremity oedema. For further diagnosis and treatment, the patient was admitted to our hospital. He had no significant past medical history.

**Fig. 1:**
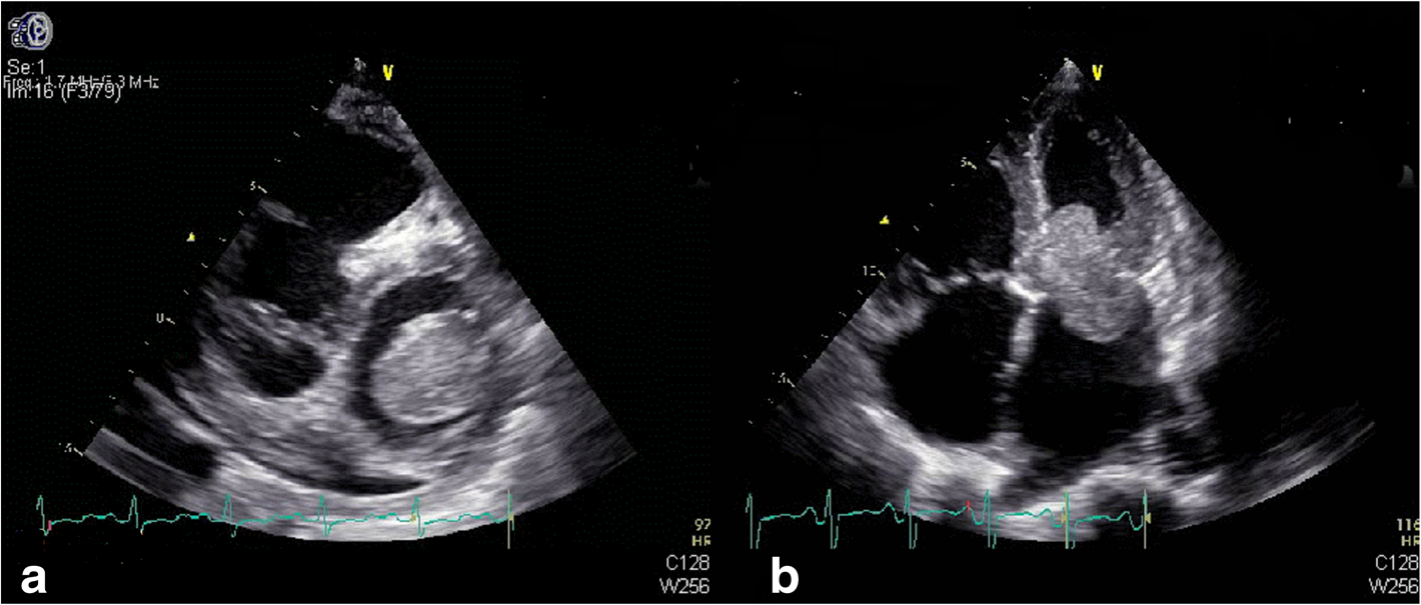
Preoperative echocardiography. **a** TTE showed a 50 × 35-mm solid tumour in the left atrium. **b** TTE showed that the tumour was adherent to the posterior annular region, obstructing the opening of the mitral valve

**Fig. 2:**
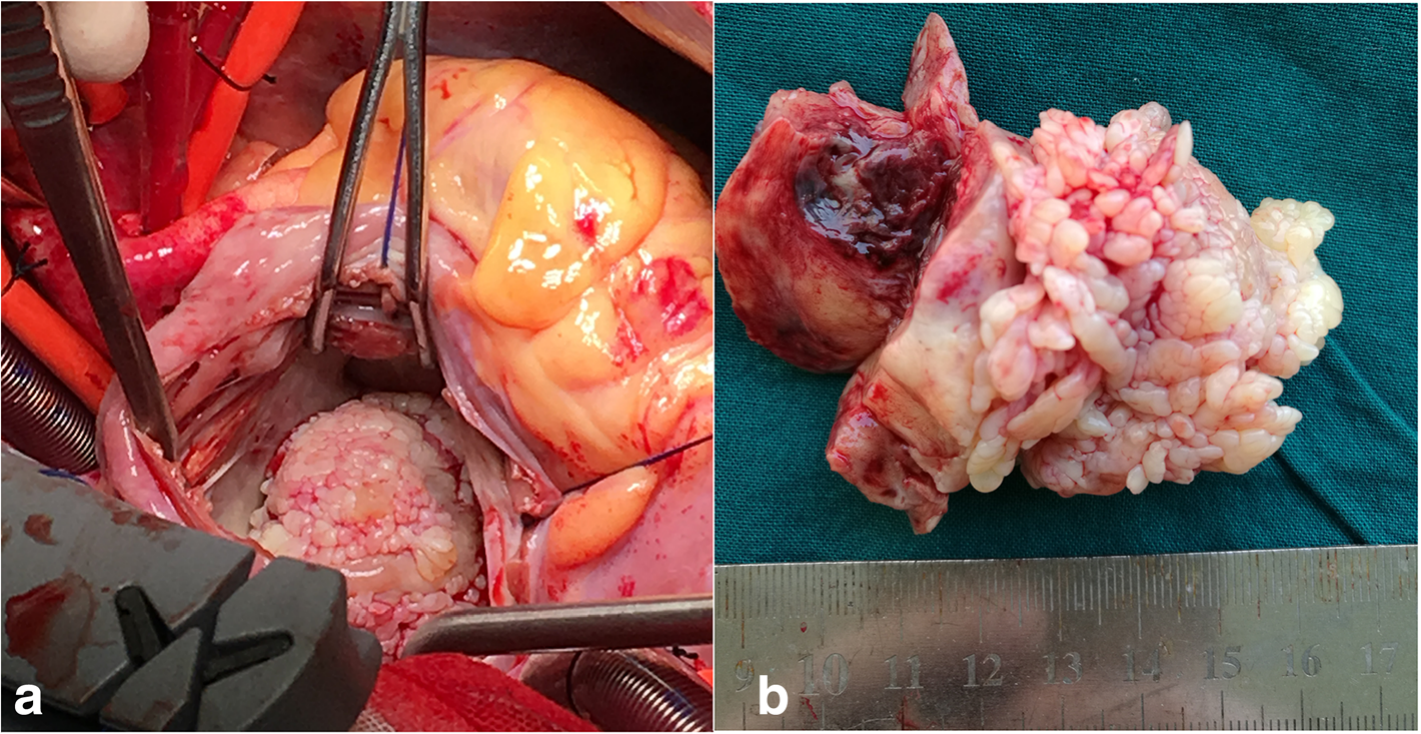
An intraoperative pictures and specimen. **a** Intraoperative picture revealed that the tumour completely fused with the mitral annulus and was divided into lobes with poly-papillary protrusions on the surface. **b** Intraoperative macroscopic findings revealed that the size of the tumour was approximately 4x6cm, and the surface was lobulated with white sea anemone-like protrusions. Sallow, fish-like tissue with cystic necrosis and haemorrhage could be seen when the tumor was cut open

**Fig. 3:**
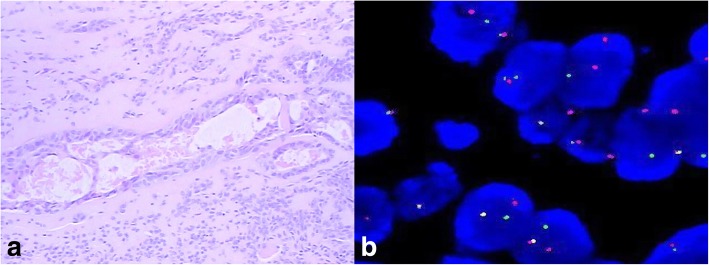
Pathology. **a** Microscopically, the tumour consists of spindle or ovoid cells and epithelioid cells (20×). **b** The result of double-colour fragmentation detection of SS18 gene probe was positive

**Fig. 4:**
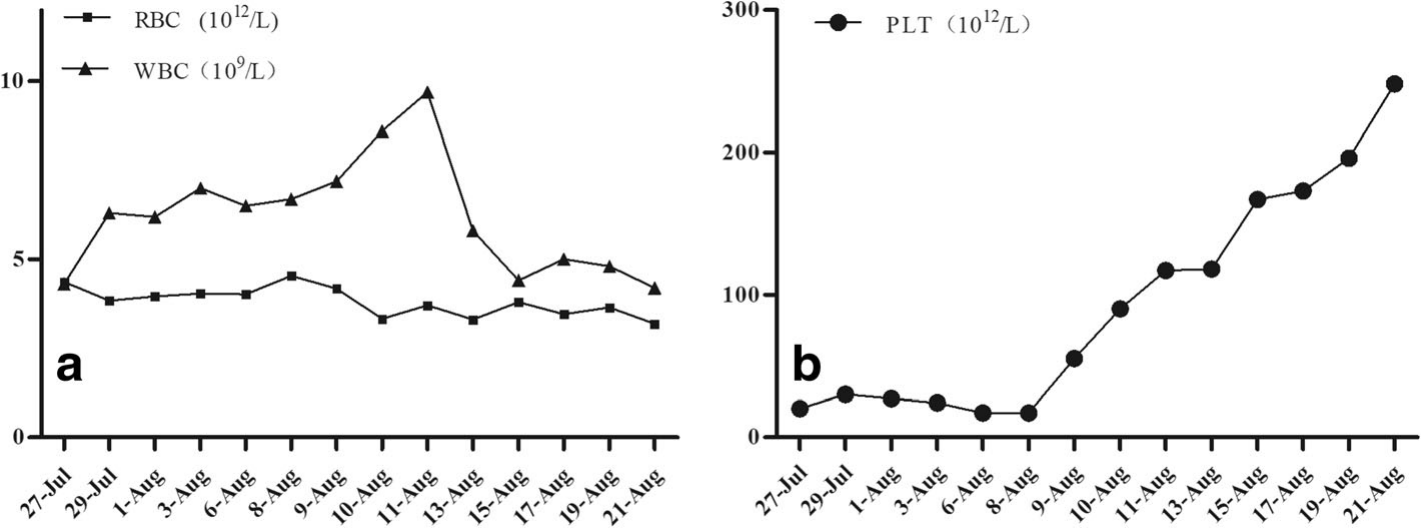
The line chart of the erythrocyte, leucocyte and the platelet counts. **a** The line chart of erythrocyte and leucocyte counts demonstrated that the counts of the erythrocyte and leucocyte were stable. **b** The line chart of the platelet counts demonstrated that the platelet counts increased after receiving 1 U platelet transfusion on Jul-28, but decreased quickly, one day before (Aug 08) the operation;2 U platelets were transfused, the platelet count returned rapidly to normal early after tumour excision without other treatment

His height was 165.0 cm, body weight was 58.1 kg, body temperature was 37 °C, pulse was 110 beats/min, blood pressure was 110/ 60 mmHg, and SpO2 was 100% (room air). Pulmonary sounds were clear with no crackles, but a III/IV systolic murmur could be heard at the junction between the left clavicle midline and the fifth intercostal space. Leg oedema was present. A chest X-ray demonstrated a cardiothoracic ratio of 60% with slight cardiac left dilation. Electrocardiography showed a sinus rhythm with a heart rate of 108 beats/min with slight ST-T segment changes. Abdominal ultrasound showed uniform congestive hepatomegaly with a normal sized spleen. Colour Doppler ruled out deep vein thrombus in the abdomen or lower limbs. A 50 × 35-mm solid mass severely adherent to the posterior part of the mitral valve was found by TTE, with systo-diastolic fluttering. The mass moved through the mitral orifice, which led to increased mitral inflow velocity but not a significant regurgitation. (Fig. 1a-b). Blood analysis revealed the following: leukocyte count of 4.3 × 10^9^/L, haemoglobin (Hb) 13.2 g/dL, platelet (Plt) count of 20 × 10^9^/L. Blood coagulation analysis revealed: Prothrombin time (14.5 s), Prothrombin activity (66%), Fibrinogen(91 mg/dL), Fibrin degradation products (30.5 μg/ml), and D-dimmer (1877 ng/ml). Blood film was performed and showed no abnormalities of platelets, leukocytes and erythrocytes. Bone marrow study revealed that the number of megakaryocytes increased; G-band and biopsy results had no abnormalities. Antinuclear antibody, Anti-ENA Antibody-Sm, Anti-ENA Antibody-RNP, Anti-ENA Antibody-SSA, Anti-ENA Antibody-SSB, Ro-52, Mitochondrial antibody IgG M2, Anti-myeloperoxidase antibody, Anti-protease 3 antibody, Anti-endothelial cell antibody and Anticardiolipin antibody were all negative. Anti-systemic lupus erythaematosus (SLE) antibodies and antiplatelet factor 4 (PF4) antibodies were also negative. Because severe thrombocytopenia was found at the same time as cerebral infarction, neither anticoagulants nor antiplatelet drugs were used during treatment. The patient received platelet transfusion, but platelet counts decreased quickly. Although operation risk was high, the tumour resection was performed through median sternal incision. Intraoperative transesophageal echocardiography (TEE) showed that the mass was adherent to the posterior mitral annulus, obstructing the mitral orifice, which caused a severe increase of pulmonary artery pressure. Intraoperative exploration revealed that the diameter of the pulmonary artery was widened, and the ratio of diameter of the aorta to the pulmonary artery was approximately 1:2. Cardiopulmonary bypass was initiated, with ascending aortic and bicaval cannulation. Following arrest with antegrade hypothermic crystalloid cardioplegia, the left atrium was revealed by blocking the superior and inferior vena cava and opening the right atrium and atrial septum. The tumour, which was rubbery to the touch, was divided into lobes with poly-papillary protrusions on the surface, and thrombus formation was observed between lobes. The pedicle was located in the area of P2 of the posterior leaflet, completely fused with the mitral annulus and lobes (Fig. 2a). Extensive resection of the tissue around the pedicle, including the annulus tissue caused mitral valve insufficiency, mitral valve replacement was performed. After cardiac resuscitation, TEE showed that the prosthetic mitral valve works regularly, and there was no residual tumor in the left atrium. The size of the tumour was approximately about 4x6cm, and the surface was lobulated, with white, sea anemone-like protrusions. Sallow fish-like tissue with cystic necrosis and haemorrhage could be seen when the tumour was cut open (Fig. 2b). Microscopically, the tumour consisted of two obviously different components, which are spindle or ovoid cells with significant marked atypia and epithelioid cells forming gland-like structures (Fig. 3a). Mitoses and focal necrosis are were present. Immunohistochemical staining showed positivity for CK, EMA, CD99, CK5/6 and CK7, focal positivity for calretinin and WT-1, and negativity for Desmin, S-100 protein, myogenin, SMA, CD31, CD34, D2–40, Sox-10, ERG, CDX-2, CK20, TTF-1, and HBME1. The Ki-67 index was approximately 10%. The result of double- colour fragmentation detection of SS18 gene probe was positive (Fig. 3b). These findings suggested that the tumour was a biphasic synovial sarcoma. The platelet count returned rapidly to normal early after tumour excision without other treatment (Fig. 4b). The results of blood coagulation analysis of the third day after surgery was significantly improved over preoperative results: prothrombin time (12.8 s), prothrombin activity (78%), fibrinogen (400 mg/dL), D-dimmer (835 ng/ml). Extubation was performed 10 h after surgery, and the patient was transferred to a general ward 2 days after surgery. The disappearance of the tumour from the annular region was confirmed on TTE 6 days after surgery, and an FDG-PET scan performed 8 days after surgery showed no abnormal accumulation. Our centre has no experience in radiotherapy and chemotherapy for cardiac synovial sarcoma. Then, we read the relevant literatures and consulted the oncologists about treatment and prognosis of synovial sarcoma. When the patient and family members were informed that even with chemotherapy and radiotherapy, the prognosis was poor, they finally decided to stop treatment. The patient was discharged when he was able to independently walk 10 days after surgery. Unfortunately, the patient died suddenly for unknown reasons 6 months later.

## Discussion and conclusion

Primary cardiac synovial sarcomas are exceedingly rare, accounting for 4.2% of cardiac sarcomas [6]. Synovial sarcomas usually occur in the soft tissues of the extremities of young adults and adolescents [7]. Their occurrence in the myocardium is deemed a rare entity. There is a male preponderance of 2.5:1. The right side of the heart is the most frequent location for cardiac synovial sarcomas, but these tumours can occur in the left side of the heart and in the pericardium as well [8]. PCSS are classified into three types: the biphasic type, which is composed of an epithelial component and a spindle-cell component, the monophasic type, which consists of only a spindle-cell component and the poorly differentiated type, which consists of small round cells [9]. These tumour often cause death through widespread infiltration into the myocardium or obstruction of the flow within the heart. There is a lack of guidelines for the treatment of PCSS, owing to the scarcity and heterogeneity of clinical data, which are derived from a limited number of cases reported, and those are mainly single case reports. Complete resection is the best treatment choice, while complete surgical resection may not be feasible in many cases due to its location [5]. In 33% of patients, complete surgical resection is possible; however, in those with apparent complete excision, high recurrence rates have been reported [10]. In a recent review that included 60 PCSS patients derived from 54 articles present in the literature, Wang et al. found that adjunctive chemotherapy could significantly prolong overall patient survival, which was rarely demonstrated in other PCSS reports [6]. Due to different adjuvant therapy options (ifosfamide/mesna, epirubicin/ifosfamide, cisplatinum/docexatel, etc.) in the few cases described in the literature, it is difficult to establish standard chemo-radiotherapy guidelines; however, doxorubicin- and ifosfamide-based chemotherapy was commonly used [6]. Siebenmann R et al. reported that PCSS can be treated by heart transplantation, and Antonella Coli et al. reported that total artificial heart implantation could offer a temporary solution prior to heart transplantation [5, 11]. In a recent extensive analysis of 60 PCSS patients, Wang et al. found that the median overall survival of the patients diagnosed with antemortem was approximately 24 months. In our case, the patient was diagnosed with atrial myxoma with severe thrombocytopenia on admission, while postoperative pathological diagnosis was biphasic PCSS, which is composed of an epithelial and a spindle-cell component. Unfortunately, the patient died suddenly 6 months later. The specific cause of death is unknown, which is a shortcoming of our case, in addition to a lack of complete follow-up.

Cardiac tumours produce a large variety of symptoms through any of 4 mechanisms. Their mass can obstruct intracardiac blood flow or interfere with valve function. Local invasion can lead to arrhythmias or pericardial effusions with tamponade. Bits of tumour can embolize, causing systemic deficits, when the tumours are on the left side of the heart. Finally, the tumours may cause systemic or constitutional symptoms. Some tumours, of course, produce no symptoms and become evident as incidental findings [3]. A prominent finding of our case was the severe thrombocytopenia, which has very rarely been reported in association with cardiac tumours, either benign or malignant [3, 4]. Thrombocytopenia due to an increased destruction of platelets may be caused by drugs, autoimmune destruction, disseminated intravascular coagulation (DIC), thrombotic thrombocytopenic purpura or haemorrhage with extensive transfusion. In our case impaired production is unlikely because the white and red cell counts were normal and systemic diseases were ruled out. Bone marrow study results excluded thrombocytopenia caused by haematologic malignancies. Antiplatelet drug use-related thrombocytopenia and Heparin-induced thrombocytopenia (HIT) was excluded, too. The patient had no drinking habits; alcohol-related thrombocytopenia was not considered. Splenic sequestration was ruled out by the normal size spleen. Normal lactate dehydrogenase and normal haptoglobin levels excluded haemolysis. Coomb’s test was negative. A negative anti-systemic lupus erythmatosus (SLE) antibodies report excluded Evans syndrome. The results of coagulation analysis strongly indicated DIC; however, that there was more than a 1 year history of severe thrombocytopenia, and no deep venous thrombosis evidence, did not support this. Idiopathic thrombocytopenic purpura (ITP) is a common cause of thrombocytopenia [12]. Most cases are considered primary ITP (80%), whereas others are attributed to coexisting conditions, such as infection with cytomegalovirus, Helicobacter pylori, hepatitis C, human immunodeficiency virus, varicella zoster, etc. [13]. Considering the marrow aspiration results, that the number of megakaryocytes increased but maturation was impaired, ITP has to be considered as the cause of thrombocytopenia. Nevertheless, the lack of evidence of end-organ damage and the lack of response to treatment with steroids and gammaglobulin did not support the ITP diagnosis. The patient received platelet transfusion, but platelet counts decreased quickly. Surprisingly, the platelet count returned rapidly to normal early after tumour excision without the other treatment. Additionally, the results of blood coagulation analysis on the third day after surgery was significantly improved compared to preoperative. An intracardiac tumour associated with thrombocytopenia is extremely rare. The most important investigations, such as biopsy or definitive surgeries, cannot be performed in patients with a significantly low platelet count; many of these cases are underdiagnosed. The exact etiopathogenesis of thrombocytopenia associated with solid cardiac neoplasms is unclear. A cardiac tumour inducing an autoimmune disorder with antibodies against target antigens on the platelets could also be considered. Sometimes mechanical forces, such as frictional and shearing stress exerted by the mere presence of intra cardiac tumours, causes significant damage to blood cells, which may be the possible explanation for this fatal thrombocytopenia. Prosthetic cardiac valves are a well-known, but ill-defined cause of thrombocytopenia. The mechanism might be similar; the tumour by its shape alters the atrial wall causing turbulence, which activates the platelets, thereby accelerating consumption. The fact that platelet counts returned to normal range early after tumour excision in our patient and in previous reports [14] supports this interpretation. In these reports, thrombocytopenia was either associated with other haematologic disorders, such as anaemia or erythrocytosis, or it was an isolated finding.

In summary, we report a rare primary cardiac synovial sarcoma case continuous with the mitral valve that caused severe thrombocytopenia; this provides further support for the awareness and diagnosis of PCSS. We also highlight that thrombocytopenia might be one rare symptom of solid cardiac tumours, but more cases are needed for support.

## Abbreviations

CT: Computed tomography
DIC: Disseminated intravascular coagulation
Hb: Haemoglobin
ITP: Idiopathic thrombocytopenic purpura
PCSS: Primary cardiac synovial sarcoma
Plt: Platelet
SLE: Systemic lupus erythmatosus
TEE: Transesophageal echocardiography
TTE: Transthoracic echocardiography
